# A case report of exudative retinal detachment derived from orbital cellulitis in mainland China

**DOI:** 10.1186/s12886-020-01596-6

**Published:** 2020-08-17

**Authors:** Wei Song, Cheng Du, Yongjie Zhang

**Affiliations:** Department of Ophthalmology, Jiaxing Hospital of Traditional Chinese Medicine, Zhongshan East Road 1501, Nanhu District, Jiaxing, 314001 Zhejiang Province PR China

**Keywords:** Exudative retinal detachment, Orbital cellulitis, Antibiotics, Corticosteroid

## Abstract

**Background:**

Orbital cellulitis is a rare cause of exudative retinal detachment. Hereby, we aimed to report the first case of exudative retinal detachment derived from orbital cellulitis in mainland China.

**Case presentation:**

A 16-year-old girl developed severe left orbital cellulitis in 4 days. Two exudative retinal detachment lesions were presented in her left eye retina. Blood cultures were performed which identified *Staphylococcus aureus*. However, the cause for the orbital cellulitis was not idenitfied in this patient. Systemic application of antibiotics together with topical antibiotics and corticosteroid was effective to the improvement of orbital cellulitis and resolution of exudative retinal detachment.

**Conclusions:**

The treatment of such clinical condition is that of orbital cellulitis in general. The exudative retinal detachment can resolve to a great extent upon cure of the underlying disease, followed by visual acuity recovery.

## Background

Orbital cellulitis (OC) is an infectious inflammation of orbital or periorbital tissues within the bony orbital cavity. Its ocular manifestations and symptoms include proptosis, erythema of eyelids, chemosis, visual acuity decline and diplopia, with or without systemic abnormalities such as fever and headache [1]. Orbital cellulitis results from bacterial infection or non-bacterial infectious organisms including fungi and viruses in adjacent tissues including paranasal sinuses, eyelids, face, or distant locations through hematogenous extension [1]. Prompt and appropriate antibiotic administration is the primary treatment for orbital cellulitis, although severe sight/life-threatening complications may still occasionally occur when antibiotics fail to clear up the infection [2].

Exudative retinal detachment (ERD) is another common ocular condition, which develops from pathological conditions that disrupt the integrity of blood-retinal barrier due to fluidic accumulation in subretinal space. Exudative retinal detachment is typically associated with inflammatory, infectious and neoplastic conditions [3], among which macular diseases such as diabetic macular edema are typical and common [4]. In this study, we report the first case of exudative retinal detachment derived from orbital cellulitis diagnosed in mainland China.

## Case presentation

A 16-year-old female patient (weight: 56 kg, height: 165 cm) was referred to ophthalmic clinic of our hospital and reported sudden left eye pain and visual acuity reduction over the past 4 days. She was febrile (38 °C) and experienced malaise.

**Fig. 1:**
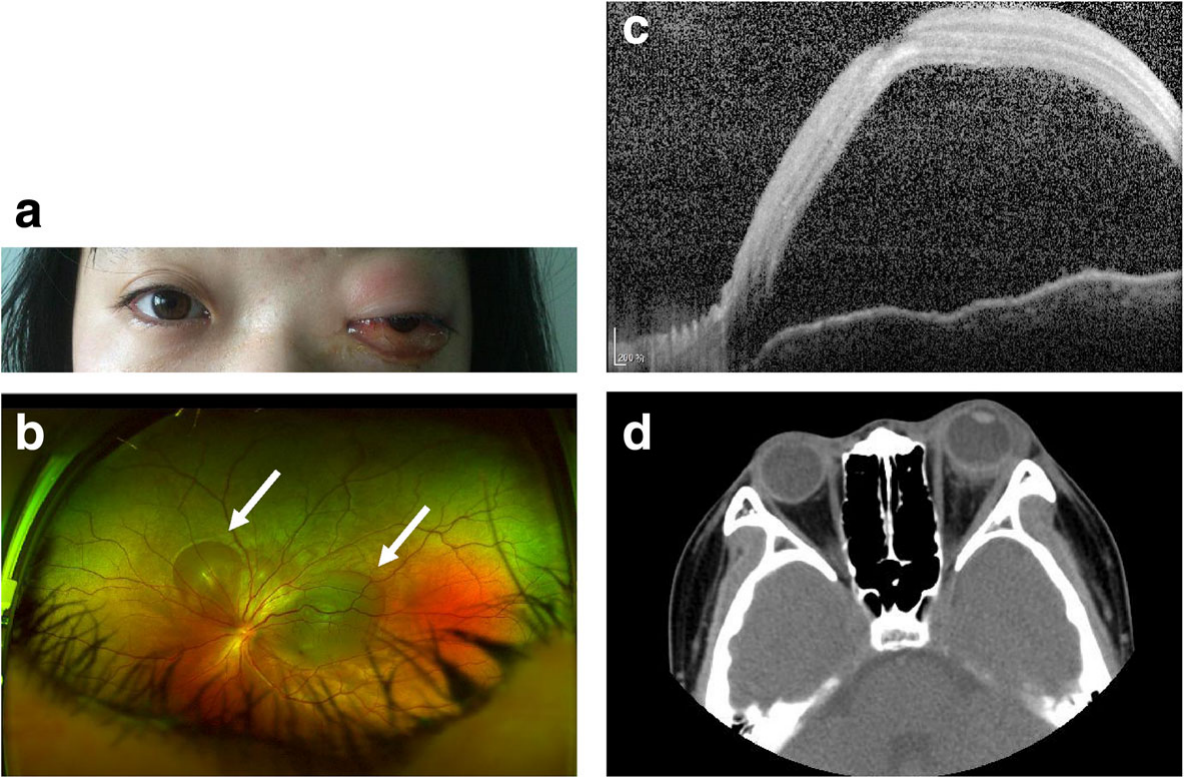
**a** The patient showed erythema and edema of the eyelids and chemosis. **b** Color fundus photograph revealed two ERD lesions that were temporal and superior-nasal to the optic disk (arrows). The presence of left eye ERD was further confirmed by SD-OCT (**c**). **d** Orbital CT scans demonstrated left eye proptosis, swelling of the periorbital and postbulbar soft tissue, and the presence of ERD (arrow)

Ophthalmic examinations revealed that the patient’s left eye visual acuity was 0.8 (LogMAR scale, no improvement was achieved after correction) and the intraocular pressure (IOP) was 18.3 mmHg (non-contact tonometer). Slit-lamp microscope showed erythema and edema of the eyelids and chemosis (Fig. 1a). Color fundus photograph revealed two ERD lesions temporal and superior-nasal to the optic disk (Fig. 1b). The presence of ERD was further confirmed by SD-OCT (spectral-domain optical coherence tomography, Heidelberg Engineering, Heidelberg, Germany) (Fig. 1c).

Systemic laboratory tests were revealed by full blood count (WBC 10.54 × 10^9^/L, 84% neutrophils) and assessment of erythrocyte sedimentation rate (ESR) (37 mm/h) and C-reactive protein (12 mg/L). Blood cultures were performed which identified *Staphylococcus aureus*. Orbital computed tomography (CT) scans identified left eye proptosis and swelling of the periorbital and postbulbar soft tissue, which all led to the diagnosis of orbital cellulitis. The presence of ERD was again verified by orbital CT scans (Fig. 1d). However, the cause for the orbital cellulitis was not idenitfied in this patient, such as trauma, periorbital cellulitis with local spread, paranasal sinusitis, or hematogenous spread of a remote infection.

**Fig. 2:**
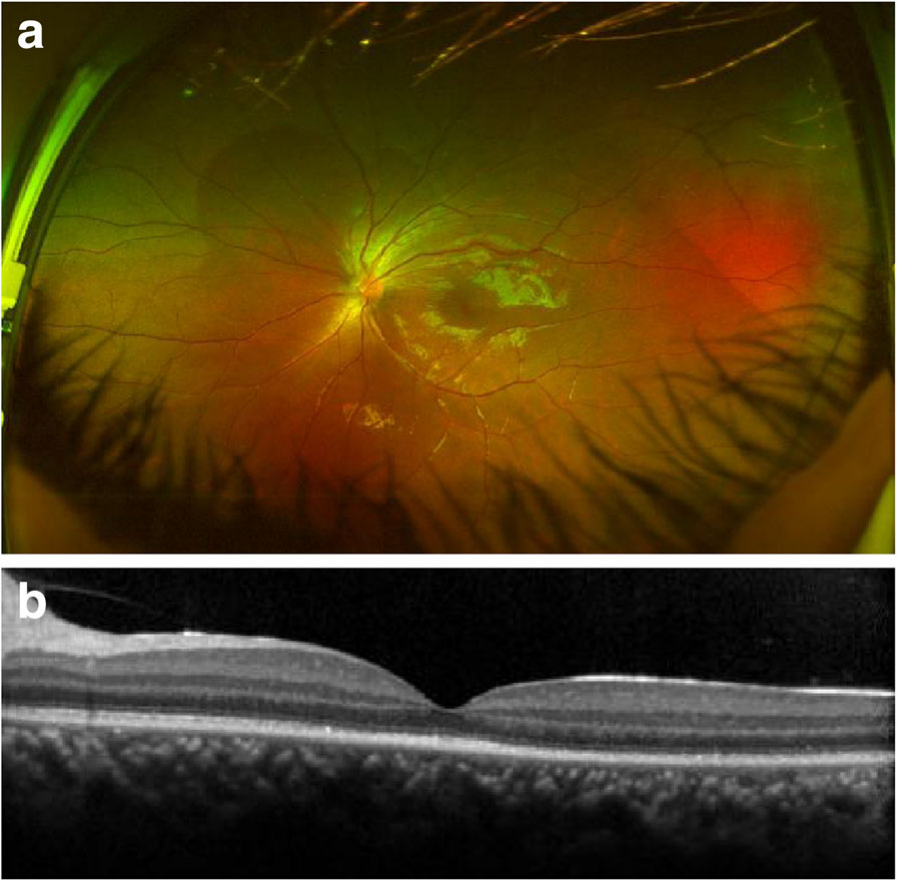
With effective therapy aimed at orbital cellulitis, complete resolution of ERD was confirmed by color fundus photograph (**a**) and SD-OCT (**b**)

Treatments in this patient were initiated soon after final diagnosis was made. Intravenous administration of antibiotics (cefatriaxone, 2.0 g, daily) was received, in addition to topical antibiotics (Lenofloxacin eye drops, Santen, Osaka, Japan) four times a day. The patient was closely followed up daily with routine ocular examinations. Considerable improvement of laboratory tests, including those revealed by the normalized blood test, was achieved after 1 week, when intravenous antibiotics were terminated. Complete recovery of ERD was evident about 1 month later (Fig. 2), and the visual acuity improved to 0.6 (best-corrected visual acuity improved to 0.4, LogMAR scale).

## Discussion and conclusions

Exudative retinal detachment can be commonly derived from noninfectious orbital inflammation such as idiopathic orbital inflammatory syndrome (IOIS), or orbital pseudotumor [3]. To the best of our knowledge, exudative retinal detachment develops from infectious orbital inflammation is an extremely rare condition worldwide. The first case was diagnosed in a 12-year-old male patient by Dr. Manmohan Malhotra in 1957 [5], To date, only two more published cases of a 56-year-old female with Down’s syndrome and an 89-year-old male with chronic myeloid leukemia were reported [6, 7].

Despite the extremely low incidence of such condition, our case further supports the development of exudative retinal detachment in the course of orbital cellulitis. The pathogenesis of exudative retinal detachment derived from orbital cellulitis may be venous congestion and reactive edema across the tough fibrous sclera [5]. Thus, no apparent hole can be visible in the retina. The essential therapy for noninfectious orbital inflammation-derived orbital cellulitis is systemic application of corticosteroids [3]. In addition, topical application of corticosteroids like intravitreal dexamethasone implant will be beneficial for such conditions including diabetic macular edema [8, 9]. However, in such rare clinical condition as exudative retinal detachment due to systemic infection of *Staphylococcus aureus*, the primary therapy should be aimed at treatment for bacteria .

In this case study, there are several shortcomings as we listed below. Firstly, the source of infection was unclear since the patient had no history of trauma, sinus infection, general infectious disorder or recent surgeries. It is widely accepted that exudative retinal detachment can be resolved to a great extent upon cure of the underlying disease, followed by visual acuity recovery. Indeed, effective treatment of orbital cellulitis resulted in rapid amelioration of the exudative retinal detachment, resembling other reported cases. Additionally, in such a condition, periocular inflammation spread to the posterior pole and may affect the optic nerve. Therefore, it is worthy to perform visual field examinations at the initiation of periocular infection attacks and at the follow-up to evaluate the effects on the optic nerve. Thirdly, since the exudative retinal detachment in this patient was derived from periocular tissues, choroidal changes were also expected to occur in such condition. However, the exudative retinal detachment occurred at the posterior pole was extremely severe as indicated in Fig. 1b and c. Thus, the condition of choroid could not be accessed by SD-OCT under the enhanced depth imaging mode.

## Data Availability

All data generated or analysed during this study are included in this published article.

## Abbreviations

CT: Computed tomography
ERD: Exudative retinal detachment
ESR: Erythrocyte sedimentation rate
IO: Intraocular pressure
OC: Orbital cellulitis
SD-OCT: Spectral-domain optical coherence tomography
WBC: White blood cell
